# New knowledge source pipelines for sociocultural representations in a cognitive architecture

**DOI:** 10.3389/fpsyg.2026.1874556

**Published:** 2026-08-10

**Authors:** Swapnika Dulam, Julian R. Gay, Christopher L. Dancy

**Affiliations:** 1 Department of Computer Science and Engineering, The Pennsylvania State University, University Park, PA, United States; 2 Department of Industrial and Manufacturing Engineering, The Pennsylvania State University, University Park, PA, United States

**Keywords:** ACT-R, cognitive architecture, cognitive model, ConceptNet, IAT, LLMs, race, sociocultural knowledge

## Abstract

**Introduction:**

With the increasing use of large language models (LLMs) and related scaled knowledge systems, it is important to achieve a better picture of the downstream impacts of the ubiquity of these systems. LLMs (as they currently are instantiated) largely output text using the skewed training data and patterns recognized through pretraining. The datasets of these systems are collected primarily from WEIRD (Western, Educated, Industrialized, Rich, Democratic) societies, and those data are often drawn from sources that represent marginalized, exploited communities (in WEIRD societies) problematically. This data process renders uneven representation, skewing potential cultural insights, and the complicated structure of LLMs can cloud the task of elucidating the full extent of problematic sociocultural structure representation within those models.

**Methods:**

Using LLMs with process-based computational cognitive models and architectures (e.g., using ACT-R) provides a potential avenue for understanding the implications of these systems on human cognitive processes. We present two systems that combine the ACT-R architecture with more scalable, holographic (vector-symbolic) memory representations – one using an LLM and another using the ConceptNet semantic network – and compare the two systems using a cognitive model of the implicit association task (IAT).

**Results:**

Though both systems are useful, we found that the ConceptNet based system more closely resembled human data from the IAT in its impact of sociocultural structure representation on behavior through memory. Nonetheless, both systems represent an opportunity to probe and evaluate those knowledge systems (i.e., ConceptNet and Llama3.2 in this case) using more cognitively relevant and plausible methods that are more generalizable to actual human behavior.

**Discussion:**

Though we present a model of a relatively simple task here in the IAT, the results point to a unique and powerful opportunity to develop a deeper understanding of what the ubiquity of these (and related) systems means for the propagation and bootstrapping of existing sociocultural knowledge and structures – especially those structures that uphold problematic, racialized representations of the human.

## 1 Introduction

With the increasing use of large language models (LLMs) and related scaled knowledge systems, it is important to understand the downstream impacts of the ubiquity of these systems. Specifically, there is a need to understand how human interaction with these systems may impact human decision-making in everyday tasks and how it may have other, potentially implicit, impacts on a person's sociocultural perspectives. Although many would consider LLM output to be biased, a more wholistic view recognizes the replication of existing sociocultural structures as the cause of such bias. When people interact with them, LLMs (and related AI systems) can perpetuate existing problematic sociocultural structures, such as anti-Black racial structures (e.g., see Dancy and Saucier, 2022), given that the constant interaction with LLMs can represent a constant cultural reinforcement (e.g., Salter et al., 2018), if not a cultural tool in itself. In many ways, this mirrors similar arguments of sociocultural replications in search engine use (e.g., Noble, 2018). Thus, understanding the degree to which this LLM replication of existing sociocultural structures impacts users' worldviews (or their own sociocultural perspectives) and the cognitive processes that mediate such replications is critical. Increased ease of use of LLMs, as well as organizational and institutional pushes for LLMs to be integrated into existing digital systems, have led to LLMs becoming an integral part of human transactive memory, a continuation of the impacts of search-engine-mediated information search on memory (Sparrow et al., 2011; Ward, 2021).

Given the potential for use of these artifacts *as* memory, there is a need to better understand how problematic social structures within LLMs might seep into human decision-making mediated by cognitive memory processes. Computational cognitive architectures such as ACT-R (Anderson, 2007) provide a useful way to understand the impact of LLMs on memory and their implications for a variety of human behaviors (Dancy and Workman, 2023), including decision making. In this article, we simulate the impact of using digital knowledge sources *as memory* by (separately) integrating an LLM and a semantic network (ConceptNet) within a cognitive architecture as a main source of knowledge for declarative memory. Despite the notably increased ubiquity of LLMs, we also test an integration of ConceptNet because of the potential advantages in using a knowledge-graph based system like ConceptNet as an alternative large knowledge base for scalable declarative memory. To focus on problematic social structures, we develop computational cognitive models of the Implicit Association Task (IAT) (Greenwald et al., 1998) and compare the impacts of the two different memory sources. This work makes several important contributions:

- A new pipeline for using large information representation systems (large language models and semantic networks) as a main knowledge source for a declarative memory system in an existing cognitive architecture.
- A comparison of using these different types of systems as knowledge sources for declarative memory.
- New methods for representing both task and socioculturally specific knowledge in one computational cognitive model.
- A new way to evaluate systems such as LLMs which is more human-centric and sensitive to impacts on human behavior at the cognitive level.

These contributions highlight this work as being important for understanding the impacts of systems such as LLMs across behavioral timescales (Newell, 1990; Lieto et al., 2018) in ways that center the human. As has been argued previously (e.g., Dancy and Saucier, 2022), exploration of the sociocultural implications of systems such as LLMs must move beyond conceptions of *bias*. Computational cognitive models represent an opportunity to explore such implications at deeper levels (Dancy and Workman, 2023) provided that the architectures that define those models' action and representational space are extended so that longer time-scale (sociocultural) structures can be adequately represented in memory at the cognitive time-scale.

## 2 Background

Though semantic networks and large language models offer great opportunities for building computational cognitive models with underlying world models in memory, an understanding of (cognitive) architectural mechanisms and their connections to scalable information representation systems such as semantic networks and LLMs is important for building such models. Connecting these more scalable potential world models to the cognitive architecture that underlies the computational cognitive model (i.e., as typical within a system that aligns with the *Standard Model of the Mind*, Laird et al., 2017, which is now more commonly referred to as the *Common Model of Cognition*) is a more generalizable way of including (potentially latent) sociocultural knowledge and structures in such cognitive models. In the proceeding sections, we give relevant background that is important for building these architecturally focused connections for the representation of sociocultural structures, beginning with background on the particular cognitive architecture we use for this work, ACT-R.

### 2.1 ACT-R

**Figure 1:**
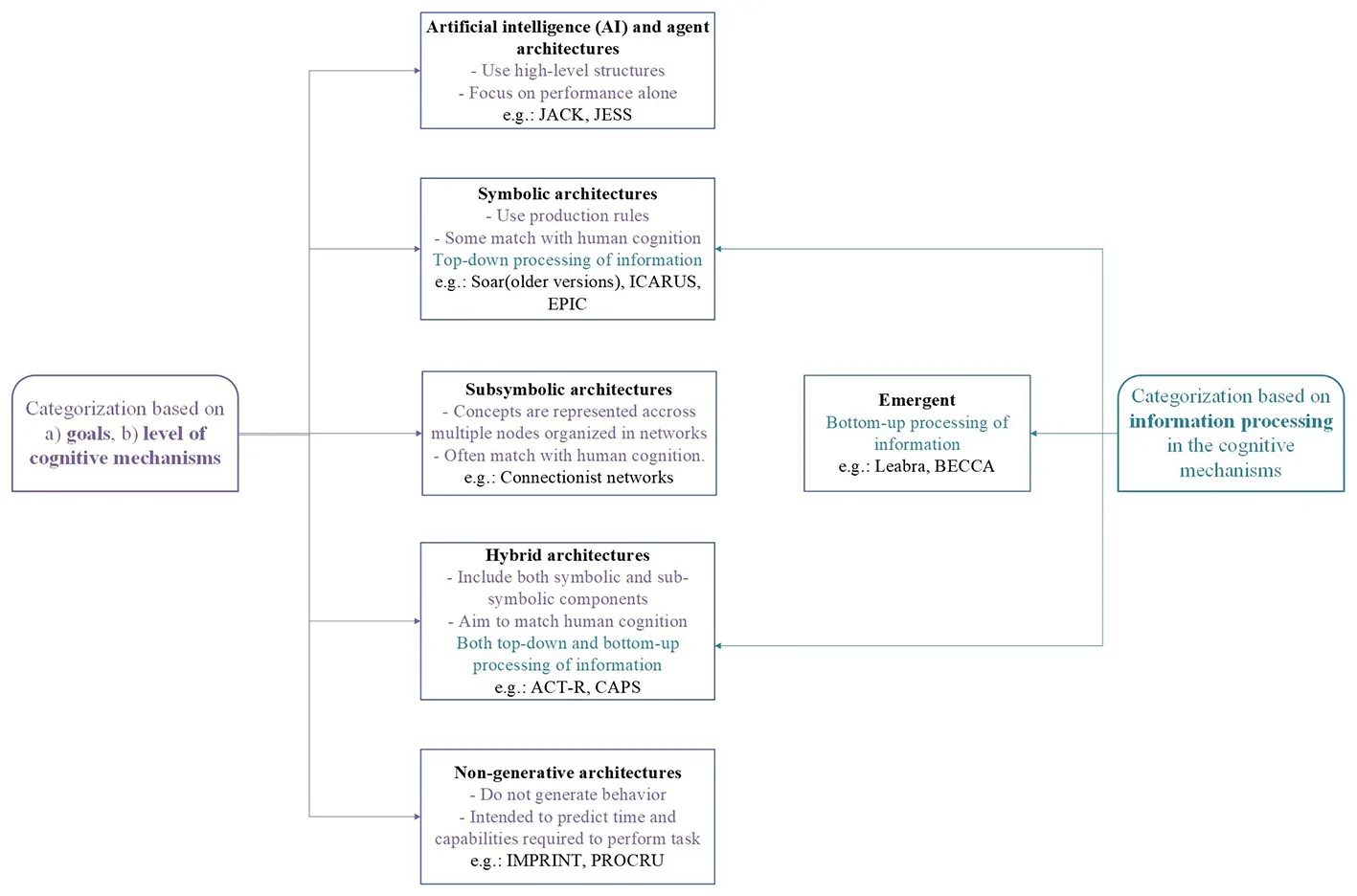
Classifications of cognitive architectures based on (Ritter et al. 2019).

Computational Cognitive architectures can be described as unified theories of cognition (Newell, 1990) implemented in software. They define the representation and action space for computational cognitive models, and (with particular models) give (information) process-oriented accounts of human behavior. There can be multiple ways to classify these architectures two of which are discussed by Ritter et al. (2019). The first is a classification based on goals of the architecture and level of their cognitive mechanisms (shown on the left of Figure 1), the second classification is based on how the information processing through these cognitive mechanisms is implemented (shown on the right of Figure 1). With the first classification just mentioned, the architectures can be classified into five main categories: a) AI or agent architectures (b) Symbolic architectures (c) Subsymbolic architectures (d) Hybrid architectures (e) Non-generative architectures. The second classification is more restrictive in the types of architectures included and can be used to classify architectures into one of three categories: a) Symbolic (b) Emergent (c) Hybrid architectures.

Adaptive Control of Thought - Rational (ACT-R) is a cognitive architecture that is classified as a hybrid architecture — ACT-R uses symbolic representation of knowledge as chunks within its declarative memory and these chunks are retrieved using sub-symbolic activation equations (Anderson, 2007). ACT-R is modular in nature, where each module represents various aspects of cognition that occur in parallel and interact with each other through a serial central procedural memory system (which acts as a serial bottleneck on many actions). ACT-R provides a theory of mapping these modules to various parts of various regions of the human brain (Anderson, 2007; Anderson et al., 2008). In the canonical implementation of ACT-R most of these modules are implemented computationally to replicate various capabilities of cognition in LISP. The mapping between the modules described in theory and their computational implementation is often not one-to-one. Each module has associated buffer(s) that help maintain the task state and act as the working memory of that module.

ACT-R modules can be categorized into two main types: memory modules and perceptual-motor modules. The memory modules include goal module, declarative module, temporal module, and an imaginal module. The goal module is responsible to maintain the current state of the ACT-R system, and helps navigate through various production rules. The imaginal module holds the temporary state of the system, acts like a scratch pad and does not persist the data like a goal module. The declarative module acts as a declarative memory system where the memories are stored as chunks with slot-value pairs. The temporal module helps in modeling time and related constraints with respect to the model. The central procedural memory system encapsulates mechanisms for action selection-related information processing and is implemented as several modules. The modules in the procedural memory system are responsible for the selection and strengthening of procedural memory, represented as production rules with utility. The production rules match the condition of the rule with the current states of the buffers and execute the related action if the conditions are met. If more than one rule's conditions match the current states of the buffers, the one with the highest utility is selected. The probability of selecting a rule and the utility of a particular rule are determined by sub-symbolic parameters within the system.

### 2.2 Vector-symbolic integrations

The cognitive models developed using ACT-R approximate human behavior in a way that is (typically) very task specific. Thus, most operate principally on a cognitive and rational behavioral time scale (Newell, 1990), and do not explicitly address the impact of larger-scale memory-based knowledge on cognitive/rational scale behavior. This typical limited time-scale of representation provided by models is likely influenced by a combination of who typically makes the models (i.e., cognitive psychologists and cognitive scientists), the tasks chosen, and the actual ACT-R declarative memory implementation, the latter of which does not scale well. Kelly et al. (2020) showed how ACT-R declarative memory's capabilities can be implemented using a distributional semantic model that can overcome the poor scalability of ACT-R's DM to big data through Holographic declarative memory (HDM, which is essentially a variant of the BEAGLE model, Jones and Mewhort, 2007).

HDM is a Vector Symbolic Architecture (VSA) in which symbols are represented as holographic reduced representations (Plate, 1995), encoding complex concepts as high-dimensional vectors rather than as discrete chunks. Specifically, it stores them as individual vectors for each feature within the chunk, and vectors from successive chunks are distributed across relevant vectors and superimposed onto previously stored information, causing vectors to gradually shift to encode better relationships among all stored chunks (Kelly et al., 2020). This distributed representation enables continuous similarity-based retrieval and partial recall, allowing HDM to efficiently store and retrieve large text corpora (Ray and Dancy, 2025). Consequently, they are able to replace the functionality of the declarative memory module in ACT-R with a vector similarity-based retrieval system.

#### 2.2.1 HDM vectors

Each *slot* of the chunk in HDM is represented by a permutation *P*_*slot*_, and each *value* is represented using two vectors: an environment vector *e*_*value*_, representing the raw perceptual identity of the value, and a memory vector *m*_*value*_, which accumulates associations from experience. The environment vectors are generated by randomly sampling from a zero-mean Gaussian distribution (with a variance of 1/*k* with *k* dimensionality), while each slot is associated with a fixed random permutation matrix. These permutation matrices act as role-binding operators by permuting vector dimensions, allowing identical values in different slots to yield different representations. These permuted environment vectors are then combined through circular convolution to construct cue vectors representing questions, to create memory vectors.

Here, we use the same example used by Kelly et al. (2020) to explain this in detail. A chunk that describes a **size:large color:black shape:square** in memory as one chunk in traditional DM is now replaced by three memory vectors for each of these values *m*_*black*_, *m*_*square*_, *m*_*large*_. Memory vectors are updated when a new value with a new context is added for the existing vector, as shown in the Equation 1 below for *m*_*square*_.


$$\begin{array}{l} m_{square,t}=\alpha m_{square,t-1}+q_{square} \end{array}$$ (1)

where *t* is the current time step, α is the forgetting rate, and *q*_*value*_ represents a query vector, which is calculated as shown below.

Each memory vector represents all cues that retrieve this value. So, to construct *m*_*square*_ for this chunk, we need four questions with different combinations of unknowns, all of which have square as the answer, to construct a cue vector which is subsequently used to update our memory vector as shown in Equation 1. These four questions can be represented as shown in the Equation 2 below.


$$\begin{array}{l} q_{square}=q_{shape:?}+q_{color:black\;shape:?}+q_{shape:?\;size:large}\\ \;+q_{color:black\;shape:?\;size:large} \end{array}$$ (2)

Each individual query vector is obtained by performing a bind operation on individual *P*_*slot*_ and *e*_*value*_ as shown for one such vector in the Equation 3 below.


$$\begin{array}{l} q_{color:black\;shape:?\;size:large}=\left(P_{color}e_{black}\right)*\left(P_{shape}\Phi \right)*\left(P_{size}e_{large}\right) \end{array}$$ (3)

Without explicit slots, HDM treats a chunk as an ordered sequence and encodes relative positions with the help of *P*_*before*_ instead of slot-specific permutations. Also, HDM uses skip-grams, meaning order is not important for association. An example computation for “What comes after large, black” is encoded in Equation 4.


$$\begin{array}{l} q_{large\;black?}=P_{before}\left(\left(P_{before}e_{large}\right)*e_{black}\right)*\Phi \end{array}$$ (4)

#### 2.2.2 HDM retrieval equations

In Kelly et al. (2020)'s implementation of HDM, retrieval is determined primarily by association strengths, rather than by ACT-R's traditional activation equation based on base-level learning, spreading activation, and partial matching. A retrieval cue vector is compared with candidate memory vectors, and the item with the highest cosine similarity is selected, typically only when this similarity exceeds a specified threshold value, which is a cosine similarity threshold derived from the ACT-R parameter for retrieval threshold. This cosine vector similarity *c* contributes to the activation of the chunk as shown in Equation 5.


$$\begin{array}{l} A=ln\left(\frac{C^{2}}{1-C^{2}}\right) \end{array}$$ (5)

This activation is then used directly in ACT-R's standard latency equation. The retrieval time of *T* of a chunk is calculated as shown in Equation 6.


$$\begin{array}{l} T=Fe^{-A_{i}} \end{array}$$ (6)

where *F* is the latency factor and *A*_*i*_ is the activation of the chunk.

The noise in retrievals is not injected into the activation value itself like in ACT-R. Instead, Gaussian noise is directly added to the distributed memory vectors during memory updates. This affects retrieval indirectly by altering the cosine similarities between cues and memory vectors, which in turn influence retrieval successes or failures, thereby affecting retrieval latency.


$$\begin{array}{l} m_{t}=m_{t-1}+\eta \Delta tn, \end{array}$$ (7)

where *m* is a memory vector, Δ*t* is the time in seconds since the last update of the memory vector, *n* is the random Gaussian vector, and η is the noise coefficient. If η is set to 0, no noise is added and there is no decay over time, while a larger value indicates more decay.

Though Kelly et al. (2020) showed that it is comparable to the typical (symbolic chunk, subsymbolic activation) in several tasks and models, HDM is actually developed within a Python variant of the ACT-R architecture — an important distinction given the differences that can arise in implementation due to the constraints and norms of particular programming languages. Ray and Dancy (2025) adapted HDM for the canonical LISP ACT-R system, such that previous ACT-R models can still use ACT-R with the HDM system (differences between the memory systems themselves withstanding) while also having those models influenced by a larger representation of knowledge (e.g., from a text corpora).

### 2.3 ConceptNet

Achieving human-like reasoning about everyday situations requires incorporating knowledge about the everyday world, that is, “commonsense knowledge” (Liu and Singh, 2004). Individuals gradually acquire this knowledge through lived-experience by developing mental models of the world, causality, social norms, physical constraints — it is rarely gathered explicitly from, for example, written communications. This implicitness creates a significant challenge for training LLMs, which must infer unstated assumptions suiting real-world contexts. Early efforts to formalize common sense knowledge focused on manually creating symbolic knowledge bases. For example, WordNet organizes words into synonym sets and captures semantic relationships, focusing on lexical categorization and word similarity (Miller, 1995; Fellbaum, 1998). Cyc, on the other hand, is an expert-created knowledge base that focuses on standardizing common sense for effective logical reasoning (Lenat et al., 1985; Lenat and Guha, 1989). DBPedia is another crowd-sourced community effort to extract structured information from Wikipedia Info boxes (Auer et al., 2007).

ConceptNet (Speer et al., 2017a) is a more recent effort to capture inferential knowledge about everyday events. It is a freely available large commonsense knowledge graph containing millions of 3-tuple assertions. These assertions connect nodes representing concepts through weighted edges from fixed set of relations. Originating from Open Mind Common Sense (OMCS) project (Singh et al., 2002), it was first released as a practical reasoning tool-kit that could be embedded in various applications that relied on commonsense reasoning (Liu and Singh, 2004). It has been continuously updated with new knowledge from crowd sourcing, expert created sources, which included data from OMCS (Singh et al., 2002), Open Multilingual WordNet (Bond and Foster, 2013), OpenCyc from Cyc (Lenat and Guha, 1989), a subset of DBPedia (Auer et al., 2007), and parsing Wiktionary. It's notable that two of these sources have also been explored as knowledge sources for the ACT-R cognitive architecture—Cyc (Ball et al., 2004) and DBPedia (Salvucci, 2014) – a salient early connection between the systems given the work presented in this paper.

The ConceptNet system also has available a set of semantic vectors called Numberbatch (i.e., ConceptNet Numberbatch, Speer et al., 2017a) that originates from the ConceptNet knowledge graph, but is also combined with knowledge from the word2Vec (Mikolov et al., 2013) and GloVe (Pennington et al., 2014) semantic vectors. These vectors allow for relatively quick similarity calculations between large sets of concepts. This embedding gives a vector representation that is potentially more straightforward to integrate with other vector-based knowledge approaches (e.g., for cognitive architectures or large language models or a combination of the two). Unsurprisingly, several attempts have been made in the past to combine semantic networks (and other related vector-based systems) and computational cognitive architectures.

Dancy (2022) provides early discussion of how ConceptNet can be integrated with ACT-R, specifically how sociocultural knowledge can be extracted from ConceptNet through term relatedness and how it is similar to declarative memory retrieval. Salvucci (2014) demonstrate how to integrate a Java version of ACT-R with relation tuples from DBPedia. Oltramari and Lebiere (2012) introduce the Cognitive Engine, which uses the HOMinE ontology that combines WordNet and FrameNet to interpret visual scenes. Lieto et al. (2018) points to other approaches that combine knowledge-level, semantic vector-related integrations into cognitive architectures, though they more generally focus on systems to address the social (and higher) *bands of behavior*.

### 2.4 Transformer architectures

Modern language processing demands architectures that can efficiently model relationships among words and phrases within larger linguistic contexts. Transformer architectures (Vaswani et al., 2017) were developed to address limitations in earlier sequence modeling approaches such as recurrent neural networks (RNNs) and long short-term memory networks (LSTMs). LSTMs process tokens sequentially and often struggle to capture long-range dependencies due to vanishing gradients and limited parallelization (Yu et al., 2019). In contrast, transformers eliminate recurrence entirely with self-attention. This design allows the model to capture dependencies regardless of positional distance, making it particularly effective for modeling complex linguistic structures. These properties have made transformer-based models a dominant paradigm in modern natural language processing.

Beyond architectural efficiency, transformers are particularly well-suited for capturing semantic and relational knowledge due to their ability to learn contextualized representations from large-scale corpora. Prior work has shown that distributional representations derived from language encode meaningful semantic relationships and associations between concepts (Harris, 1954; Caliskan et al., 2017). Transformer-based models extend this capability by incorporating context-dependent representations, allowing the same word to take on different meanings depending on surrounding tokens. As a result, transformers can encode nuanced associations between social groups and attributes, which can be surfaced through controlled prompting and structured extraction. These properties make them an appropriate foundation for extracting relational knowledge that can be subsequently mapped into symbolic cognitive presentations.

A transformer consists of stacked layers of multi-head self-attention and position-wise feedforward networks. Given an input sequence *x* = (*x*_1_, *x*_2_, ..., *x*_*n*_), the model computes contextualized token representations by attending to all other tokens in the sequence. Self-attention operates by projecting tokens into query, key, and value spaces, enabling the model to compute attention weights that determine the relevance of each token to others (Vaswani et al., 2017). This mechanism is particularly well-suited to our task, as sociocultural knowledge often emerges from contextual co-occurrence patterns rather than from explicit statements. When trained on a large-scale text corpora using next-token prediction objectives, transformer architectures can be scaled into large language models (LLMs). These models learn broad linguistic, semantic, and world knowledge representations from billions of tokens (Brown et al., 2020). Beyond general language generation, LLMs have started being used as sources of commonsense, world knowledge, and relational knowledge (Petroni et al., 2019; AlKhamissi et al., 2022; Cohen et al., 2023).

Recent work on integrating LLMs with ACT-R has explored hybrid architectures for decision-making in applied domains such as manufacturing (Wu et al., 2025). They proposed a pipeline in which structured cognitive traces from ACT-R are transformed into vector representations and processes by a transformer-based model to support downstream classification and decision-making tasks. ACT-R produces procedural traces that are embedded and passed through attention-based layers, where contextualized representations are formed and subsequently used to guide decisions via a classification head. This approach leverages the strengths of both symbolic and sub-symbolic paradigms: ACT-R provides interpretable, rule-based cognitive structure, while the transformer captures high-dimensional patterns for generalization (Wu et al., 2025). However, the integration primarily operates by converting symbolic outputs into vectorized inputs for neural processing, emphasizing performance and task optimization rather than preserving or reinserting structured knowledge into the cognitive architecture. In contrast, our work focuses on the inverse direction. We extract sociocultural knowledge from LLMs and encoding it into symbolic ACT-R representations, thereby enabling analysis of how such knowledge influences declarative memory processes and, by extension, decision making processes that rely on declarative memory (e.g., Gonzalez et al., 2003).

Complementary research has examined more tightly coupled integrations between LLMs and cognitive architectures, with multiple architectural configurations being proposed (Romero et al., 2023). This includes cognitively-augmented LLMs (Sumers et al., 2024; Park et al., 2023), LLM-driven perception and motor modules (Driess et al., 2023; Wang et al., 2023), and fully embedded LLM components within cognitive architecture subsystems such as working memory, declarative memory, procedural memory (Yao et al., 2022; Romero et al., 2023), integrating word similarity based on LLM embeddings within ACT-R's declarative memory equations (Meghdadi et al., 2026). These designs treat LLMs as functional components within the cognitive loop, enabling capabilities such as planning, simulation, and contextual reasoning through iterative interaction between symbolic and neural elements. Notably, the architectures emphasize bidirectional information flow, where LLM outputs influence cognitive state and vice versa. Thus, it supports more adaptive and robust behavior (Romero et al., 2023). Although this work advances the integration of neural and symbolic systems at the architectural level, it does not explicitly address how sociocultural knowledge embedded in LLMs can be systematically extracted, structured, and analyzed within a cognitive framework. Our approach complements this line of research by focusing on knowledge-level integration, transforming implicit associations within LLMs into explicit declarative memory representations that can be evaluated within ACT-R.

### 2.5 Sociocultural perspectives

Sociocultural perspectives emphasize that language reflects the social, historical, and cultural contexts in which it is produced, rather than serving as a neutral medium of communication. In natural language processing (NLP), this has led to a growing body of work examining how models encode and reproduce racial bias. Studies on racial bias in NLP have shown that language models and word embeddings systematically capture associations between racial groups and evaluative attributes, often reflecting and amplifying societal inequalities. Studies further argue that these patterns arise from both imbalances in training data and broader sociocultural structures embedded in language. From this perspective, biases in language models are not simply errors to be eliminated, but signals of underlying sociocultural knowledge (Shearer et al., 2019). In this work, we adopt this view by treating large language models as sources of extractable sociocultural associations, which can be structured and analyzed within a cognitive architecture to better understand how such knowledge is represented and influences behavior.

A large, and still growing, body of work has argued and demonstrated that language models (and related generative AI systems) encode sociocultural biases present in their training data (e.g., Bender et al., 2021) and in the AI engineering process itself (e.g., Workman and Dancy, 2026; Beyene and Dancy, 2026). In earlier work, (Caliskan et al., 2017) showed that word embeddings capture human-like associations between social groups and attributes, introducing the Word Embedding Association Test (WEAT) as a computational analog to the Implicit Association Test (IAT). This establishes that distributional representations derived from language corpora contain measurable sociocultural structure, even if those representations may be latent.

More recent studies extend these findings to generative large language models. They demonstrate that pretrained models systematically output stereotypical associations over neutral alternatives, confirming that such biases persist in modern architectures (Nadeem et al., 2021; Navigli et al., 2023). These results suggest that LLMs encode rich, through often implicit, relational knowledge about social concepts.

However, this capability raises important concerns. One argument states that LLMs reproduce patterns from their training data without true semantic understanding, potentially amplifying harmful biases and misleading users. From this perspective, the associations generated by LLMs should not be treated as neutral knowledge, but rather as reflections of underlying distributions of sociocultural structures (Bender et al., 2021) present in the full AI engineering process [from situation development to data processes to model development and beyond, as argued by Workman and Dancy (2026)].

## 3 Method

To evaluate the influence of sociocultural knowledge embedded within LLMs and semantic network representations, we developed a structured testing pipeline grounded in cognitive science. Central to the pipeline is the incorporation of a cognitive model for the implicit association task, which helps us measure latent problematic representations in the data. We provide a comprehensive description of the pipeline architecture, including the design and parameterization of the ACT-R model we developed for the IAT task, the interfacing mechanisms with LLMs and Semantic networks with the help of Holographic declarative memory (HDM), and the evaluation metrics used to quantify model behavior. Overall, the pipeline enables a structured and reproducible approach to examining how sociocultural knowledge manifests in computing systems.

### 3.1 Architecture pipeline

**Figure 2:**
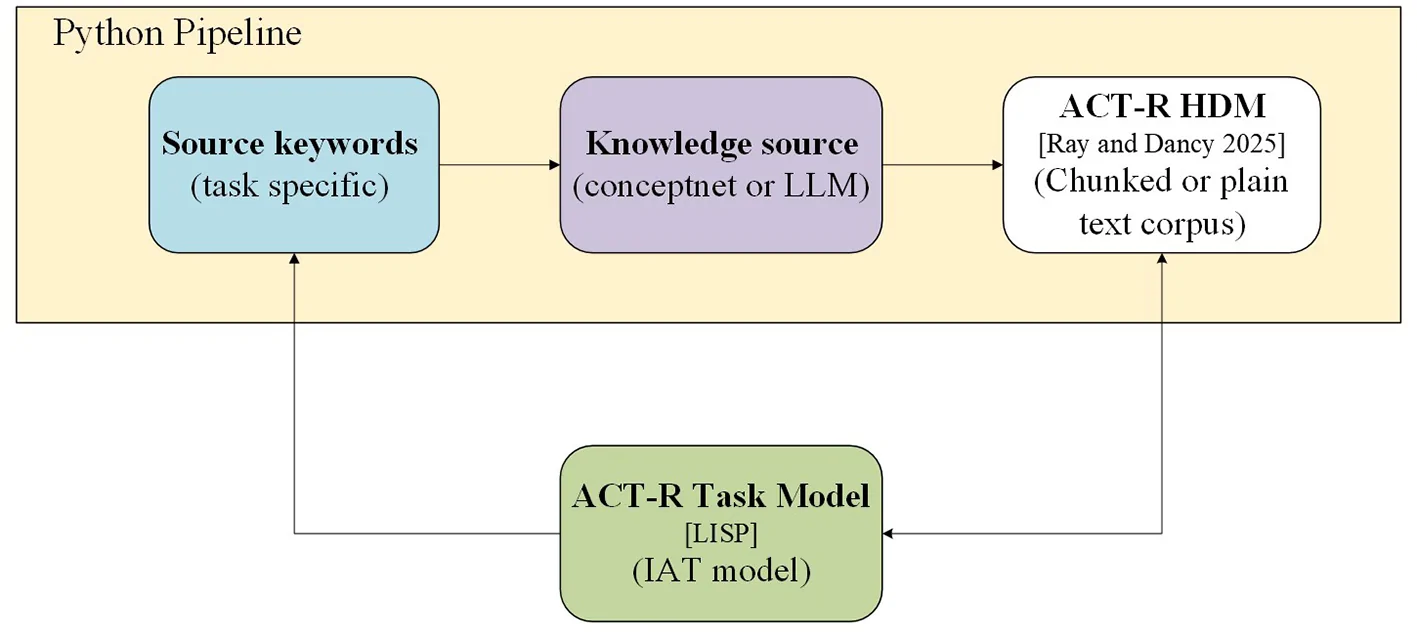
High level architecture pipeline of the task; the pipeline starts from task-specific source keywords based on ACT-R task, data curation from either source which is sent to ACT-R model through HDM.

Extending the approaches proposed in (Dancy, 2022) for representing sociocultural knowledge from knowledge graphs with ACT-R, we integrate these systems through the proposed pipeline, as shown in Figure 2. Following the method suggested by (Salvucci, 2014), task-relevant declarative chunks are stored in memory, while broader world knowledge is sourced from external systems such as ConceptNet or Llama. To support this integration, we replace ACT-R's declarative memory module with a holographic declarative memory module as described in Ray and Dancy (2025).

We begin by identifying the source data required for the task, which in the current case is the IAT to construct a dataset of keywords presented to the participants. This dataset, consisting of approximately 30 keywords, is then used to query an external knowledge source, generating additional text semantically related to the original terms. This step is intended to extract task-relevant information from a source that encodes a broader sociocultural context. The resulting generated corpus is subsequently encoded within ACT-R's declarative memory. Finally, we execute the ACT-R model using this enriched corpus and collect output data to compute relevant measures.

### 3.2 IAT task

A task that uses latent representation of sociocultural knowledge is useful to test the cognitive model at a social band level. In our approach, we narrow the broader domain of sociocultural knowledge to the more specific domain of racialized knowledge representation. For this purpose, we adopt the Implicit Association Test (IAT) (Greenwald et al., 1998) as the experimental paradigm for assessing the architecture. The IAT is particularly well-suited for this objective, as it captures implicit associations through differential response patterns, enabling us to probe representations that may not be explicitly accessible. Nevertheless, the race IAT can be swapped for a different IAT to explore distinct latent associations.

IAT uses latency-based measures such as response times to evaluate implicit associations between certain concepts. We use the race-based IAT implementation from (Peirce et al., 2019) as our source to test our ACT-R model for the IAT task. In this task, pleasant (represented through keywords joy, pleasure, happy, love, and good) or non-pleasant attributes (represented through keywords bad, agony, evil, hurt, and nasty) are associated with images of Black or White people. The stimuli are presented in 5 blocks, with blocks 1, 2, and 4 being train and blocks 3 and 5 being test. The train blocks have 10 trials each, and the test blocks have 20 trials each, all presented in random order within each block. The first block presents stimuli related to pleasant mapped with the “a” key on the keyboard and non-pleasant attributes mapped to the “l” key on the keyboard. In the second block, which is the congruent train condition, pictures of White and Black individuals are displayed in random order and are mapped to the keys “a” and “l”, respectively. In the third block, which is the congruent test condition, the pictures of White individuals and keywords associated with pleasant stimuli are mapped to the “a” key, and those of Black individuals and unpleasant words are mapped to the “l” key. For the next block, which serves as training for the incongruent condition, the key mapping for Black and White stimuli is switched so that Black and White stimuli map to opposite keys. In the final round, which tested mapping under incongruent conditions, pictures of Black individuals and pleasant keywords were mapped to the “a” key, and pictures of White individuals and unpleasant keywords were mapped to the “l” key.

The ACT-R IAT task used ACT-R GUI's experiment window to simulate the visual inputs that are displayed on the screen. The ACT-R model used production rules that used chunks from declarative memory to map the inputs displayed on the screen to their corresponding keys to be pressed. The ACT-R task collected response times and key presses for the task. IAT has a *d* − *score* metric, calculated as the difference in mean response latencies between congruent and incongruent blocks, divided by the pooled standard deviation of those latencies (Greenwald et al., 2003). A value closer to 0 indicates no implicit bias; the farther the value is from 0, the more strongly there exists a preference for one group over the other, with a positive score indicating a higher preference for the (racialized) congruent condition and a negative score for the incongruent condition, here a negative score represents an implicit association between Black and pleasant.

### 3.3 ACT-R model

The ACT-R model for the IAT task was developed using the canonical (Lisp) version of ACT-R. The model for this task utilized declarative memory to store the chunks relevant to the task, used the vision module to attend to the inputs, and used a motor module to initiate key press actions using a virtual keyboard. The declarative memory chunks had slots for probe, race, and valence. The model began each trial in an initial state, attended to the visual input, encoded the current block information, and issued a retrieval request. Based on the retrieved chunk and the available task information, it selected and pressed the corresponding response key. If retrieval failed, returned an incorrect chunk, or no chunk satisfied the retrieval criteria, the model pressed a dummy key to terminate the current trial and proceed to the next one. Each subsequent trial restarted from the initial state, and this process continued until all trials had been completed. To speed up the model execution time, the latency factor parameter, :lf, was set to 0.2. This model is represented by a green box in Figure 2. This ACT-R model was then integrated with holographic declarative memory (HDM) (Ray and Dancy, 2025) represented by a white box in Figure 2 and our pipeline in Python (remaining blue and purple boxes).

The HDM adaptation for Lisp ACT-R (Ray and Dancy, 2025), ensured that our existing IAT model was able to run with the Holographic declarative memory replacing the declarative memory module of ACT-R. The parameters such as noise and threshold were kept at the default parameters from Kelly et al. (2020)'s implementation. This basic integration is what we considered “Baseline” for our evaluations. The HDM adaptation allows for a large corpus of text to be easily added as the declarative memory for the ACT-R model. We tried two different methods to add a large corpus to HDM. For the first method, we followed the approach suggested by Ray and Dancy (2025) to add the text file itself. We used this ability to test different sources (ConceptNet, LLM) of the corpus and analyze their impacts on the model. For the second method, we used automated chunk creation where the statement triplets were directly added to HDM following the chunk creation format in ACT-R.

### 3.4 Text Corpus as HDM input

When using an entire text corpus as input to HDM, the corpus must first be preprocessed to remove *stopwords*. This step is important, without which more noise gets added to the chunk vector construction, and the number of records retrieved correctly would be low. After preprocessing the input file, the generated file is sent as input to the HDM module. For every line in the input corpus, HDM stores the associations of the chunks in left-to-right order (Ray and Dancy, 2025). For each data source, ConceptNet and LLM, we used three different sets of input keywords (*seed words*) to generate our corpus. The first set contains all terms exposed to the user along with terms describing both races, hereby referred to as *All*. The next set was built by removing all White race-related terms from the *All* set, hereby referred to as *Black*. Finally, the third set was built by removing all Black race-related terms from the *All* set, hereby referred to as *White*. A detailed list of the seed words along with stats related to generated sentences with a representative example is given in Table 1.

**Table 1:** Seed words used for ConceptNet and LLM and their stats.

Word	# of sen	Total # of raw words	Total # of pp words	Avg words per sen	Avg pp words per sen	Cos sim	Example
Race [CN]	24	146	82	6.08	3.42	0.71	race is related to running
Race [LLM]	13	57	31	4.38	2.38	0.8	Race is a competition.
Ethnicity [CN]	1	6	3	6	3	0.78	ethnicity is a type of quality
Ethnicity [LLM]	31	152	95	4.9	3.06	0.7	Ethnicity relates to culture.
(B) Black [CN]	33	181	103	5.48	3.12	0.72	black is related to dark
(B) Black [LLM]	24	148	74	6.17	3.08	0.81	Black is related to darkness.
(B) African_american [CN]	2	15	9	7.5	4.5	0.81	African-American is a synonym of Afro-American
(B) African_american [LLM]	27	158	110	5.85	4.07	0.68	African Americans are similar to blacks.
(B) African [CN]	53	321	162	6.06	3.06	0.55	African is a type of person
(B) African [LLM]	31	150	93	4.84	3	0.56	Africa is a continent.
(B) Black_man [CN]	1	7	3	7	3	0.7	a black man can do this better
(B) Black_man [LLM]	29	171	109	5.9	3.76	0.74	Black man is a human.
(B) Black_person [LLM]	29	143	99	4.93	3.41	1	Black person is a race.
(B) Black_african [CN]	2	14	8	7	4	0.67	Black African is a type of African
(B) Black_african [LLM]	12	57	43	4.75	3.58	0.74	Black Africans are Africans.
(B) Afro-american [LLM]	26	160	107	6.15	4.12	1	Afro-Americans are black.
(B) Female_black_person [LLM]	14	91	68	6.5	4.86	1	Female Black people can vote.
(W) White [CN]	42	225	127	5.36	3.02	0.65	white is a type of man
(W) White [LLM]	12	61	33	5.08	2.75	0.77	White is a color.
(W) European_american [LLM]	17	93	68	5.47	4	1	European Americans are similar to Americans.
(W) Caucasian [CN]	9	57	30	6.33	3.33	0.69	Caucasian is a synonym of White person
(W) Caucasian [LLM]	32	180	109	5.63	3.41	0.74	Caucasians are Indo-European.
(W) White_woman [CN]	2	15	9	7.5	4.5	0.81	white woman is a type of woman
(W) White_woman [LLM]	29	167	117	5.76	4.03	0.73	White women are Caucasians.
(W) White_man [CN]	4	32	20	8	5	0.77	white man is a type of man
(W) White_man [LLM]	28	165	108	5.89	3.86	0.79	White man is Caucasian.
Human [CN]	71	521	243	7.34	3.42	0.43	A human has muscles
Human [LLM]	28	107	77	3.82	2.75	0.61	Humans are mammals.
Good [CN]	41	203	117	4.95	2.85	0.64	good is related to better
Good [LLM]	21	102	60	4.86	2.86	0.75	Good can be praised.
Happy [CN]	42	277	149	6.6	3.55	0.71	happy is related to emotion
Happy [LLM]	32	147	90	4.59	2.81	0.83	Happy is related to joy.
Joy [CN]	23	131	80	5.7	3.48	0.74	joy is related to happiness
Joy [LLM]	34	168	94	4.94	2.76	0.83	Joy causes smiling.
Love [CN]	44	293	167	6.66	3.8	0.65	love is a feeling
love [LLM]	35	171	100	4.89	2.86	0.76	Love is related to feelings.
Pleasure [CN]	30	178	102	5.93	3.4	0.67	music is for pleasure
Pleasure [LLM]	30	127	78	4.23	2.6	0.82	Pleasure relates to enjoyment.
Bad [CN]	54	276	158	5.11	2.93	0.64	bad is related to unfavorable
Bad [LLM]	30	186	109	6.2	3.63	0.78	Bad is related to evil.
Agony [CN]	8	47	24	5.88	3	0.81	agony is a type of suffering
Agony [LLM]	30	137	82	4.57	2.73	0.88	Agony is a pain.
Nasty [CN]	15	79	45	5.27	3	0.77	nasty is similar to difficult
Nasty [LLM]	31	138	86	4.45	2.77	0.78	Nasty relates to unpleasant.
Evil [CN]	19	104	58	5.47	3.05	0.73	evil is related to wicked
Evil [LLM]	29	148	88	5.1	3.03	0.79	Evil is related to morality.
Hurt [CN]	19	117	61	6.16	3.21	0.61	hurt is a synonym of distress
Hurt [LLM]	28	114	65	4.07	2.32	0.69	Hurt is an injury.
Positive [CN]	26	150	84	5.77	3.23	0.54	positive is similar to advantageous
Positive [LLM]	17	86	61	5.06	3.59	0.66	Positive emotions are good feelings.
Negative [CN]	25	141	79	5.64	3.16	0.7	negative is similar to antagonistic
Negative [LLM]	29	129	74	4.45	2.55	0.74	Negative is a concept.

Specifically, the number of sentences per seed word, the total number of words per seed word (raw), the total number of words per seed word after preprocessing, the average words per sentence (raw), the average words per sentence (preprocessed), average cosine similarity of the sentences from the current corpus (CN or LLM) that match the sentences in the other corpus(LLM or CN respectively) and an example of a sentence for each seed word. Seed words black_person, afro-american, female_black_person, european_american did not yield any sentences from ConceptNet. A higher cosine similarity indicates that similar sentences were generated and that LLM responses are not hallucinated. While this metric is to address the factual correctness of data generated by LLM, this by no means cover exhaustive facts and that different sentences generated from one source can match with highest similarity to only one sentence in the other source.

#### 3.4.1 ConceptNet

**Figure 3:**
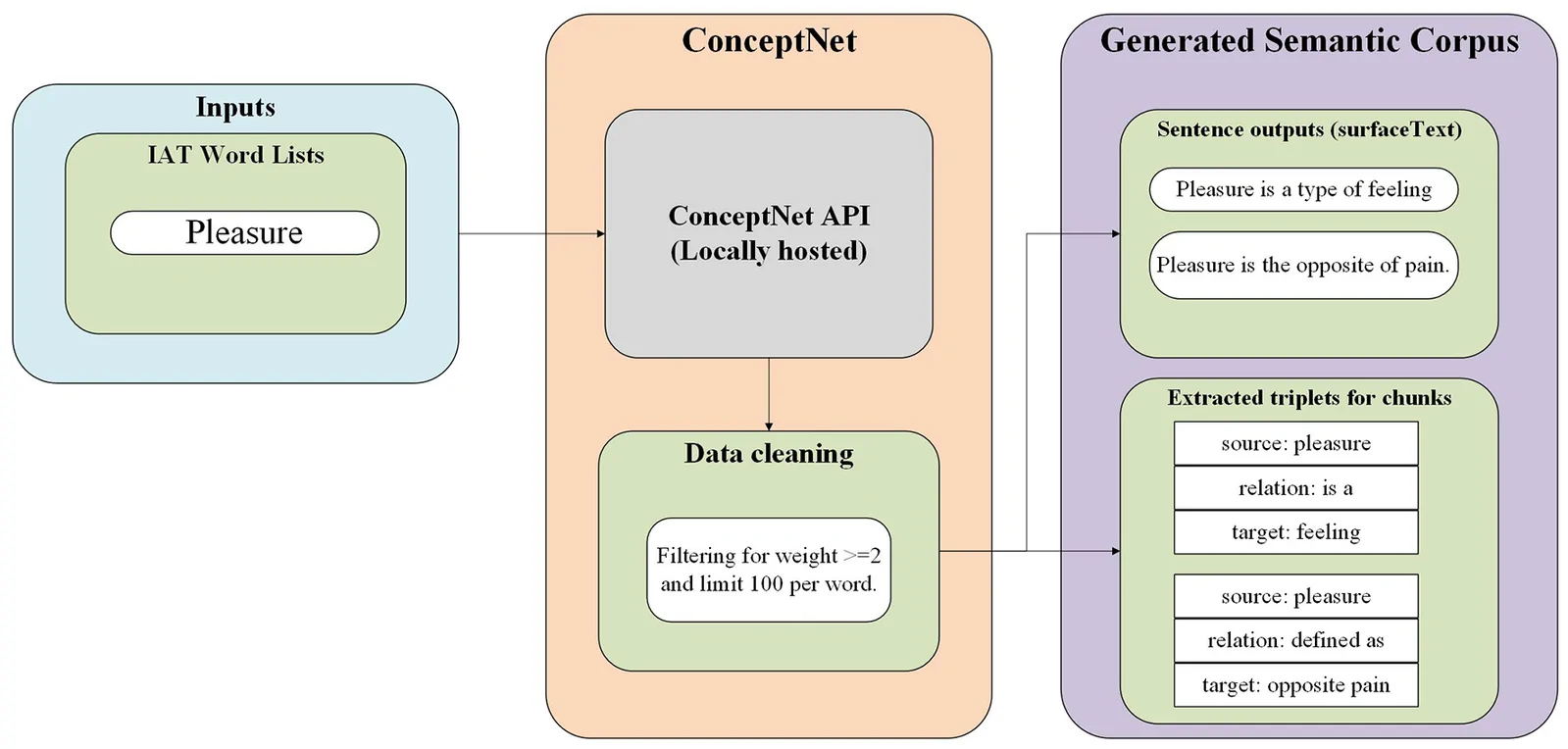
Pipeline for generating a semantic corpus from IAT word lists using ConceptNet API, producing relational and sentence-based representations of concept associations for both text and chunked inputs.

We built a local ConceptNet instance from (Speer et al., 2017b). We then used a local API to access the relations to a particular concept. We use the terms (*seed words*) from the source dataset and query this API to build our dataset for the task. We set a limit of 100 related terms per concept and selected terms in English with a weight of at least 2.0 to eliminate noisy associations. We used the “SurfaceText” attribute from the API response. The “SurfaceText” attribute uses natural language to form a sentence with the source entity, its relation to the target entity, and the target entity itself. For example, for *source entity: “Afro-American,” relation: “SimilarTo,” target entity: “Black” has a “surfaceText”: “Afro-American is similar to Black”*. Any given seed word could be either a source or target entity. Given the directional nature of much of the relations in ConceptNet (and the details of the process), this process did not result in any identical sentences between seed words. The sentence-like structures produced by this process fit well for use by the implementation of HDM by Ray and Dancy (2025), which expects a text corpus. A detailed extraction process is shown in Figure 3.

As described, we collect the sentences from predefined sets of seed words (which themselves are described in the previous section.) Based on this process we had in the range of 500–600 sentences, which form our input text corpus to HDM. Table 2 gives a high-level view of the corpus created by each set of seed words (with the *Black* and *White* sets of seed words being subsets of the *All* set). Given our process and the Conceptnet system (and the relational density of some concepts within the Conceptnet system), the distribution of sentences amongst seed words was not uniform; some words (e.g., human) had a higher sentence count. As discussed in the next section, the differences between the Conceptnet and LLM method of corpus generation resulted in a different word and sentence profile within the corpus.

**Table 2:** ConceptNet corpora level stats.

Word	Total # of sen	Total # of raw words	Total # of pp words	Avg words per sen	Avg pp words per sen
All	590	3536	1923	6.2	3.4
Black	533	3207	1737	6.1	3.3
White	499	2998	1638	6.1	3.4

Specifically, the total number of sentences for each corpus, the total number of words for each corpus, the total number of words after preprocessing for each corpus, the average words per sentence (raw), the average words per sentence (preprocessed).

#### 3.4.2 LLM: Llama 3.2

**Figure 4:**
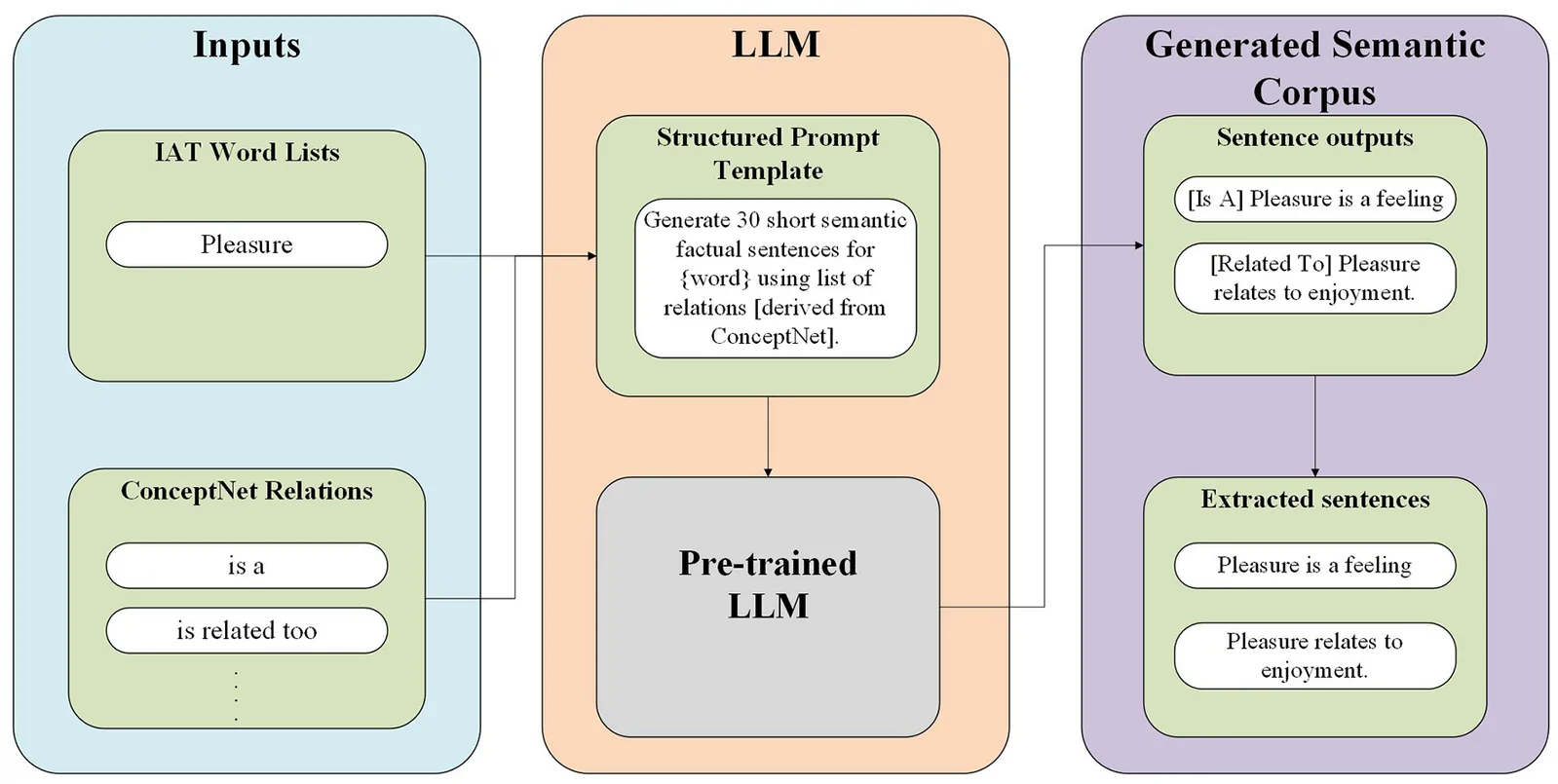
Pipeline for generating a semantic corpus from IAT word lists using a structured prompt and a pre-trained LLM, producing relational and sentence-based representations of concept associations.

Large language models (LLMs) serve as the primary mechanism for generating sociocultural knowledge alongside ConceptNet in our framework. Similar to ConceptNet, the model is given a set of input terms and prompted to produce semantically meaningful sentences and relational expressions that capture associations between concepts. This process leverages transformer-based models' ability to encode rich contextual and semantic knowledge from large-scale text corpora (Vaswani et al., 2017). Prior work has shown that such representations capture implicit associations between social groups and attributes, reflecting underlying sociocultural patterns present in language (Caliskan et al., 2017). As illustrated in Figure 4, the LLM transforms structured prompts into a generated semantic corpus consisting of both group-related statements and general sentence outputs. In our pipeline, this corpus serves as an intermediate representation that is subsequently converted into ACT-R-compatible chunks for cognitive modeling and analysis.

We specifically utilize a transformer-based large language model (Meta's Llama 3.2 3B Instruct, accessed via the Hugging Face Transformers framework) to generate a corpus of relational knowledge from the *seed words* list. The model is loaded using an autoregressive causal language modeling interface (AutoModelForCasualLM) with automatic device mapping (device_map=‘auto') and mixed-precision inference. Rather than querying an external knowledge base, we provide the full set of input words to the model using a structured prompt designed to elicit relational triplets and supporting sentences. Specifically, a prompt template is input into the model to generate outputs in the form [*relation, sentence*], the sentences here express the relationship with the queried source.

To ensure a cognitively meaningful and factually accurate extraction of sociocultural knowledge, we define a structured schema inspired by ‘LMCRAWL' Cohen et al. (2023) that represents knowledge along with the relation to the source entity, consistent with ConceptNet. While, the first step in ‘LMCRAWL' is generating relations for the given entity, we instead used the relevant relations from ConceptNet such as “Is A,” “Related To,” “Similar To,” “Capable Of,” “Part Of,” “Used For,” “Causes,” “Defined As,” “Derived From,” “Form Of,” and “Instance Of,” as input, to generate pure sentences. Additionally, we used the “don't know” sentence generation strategy if a fact connecting a relation to the source entitity does not exist to ensure factual correctness of data generated. This schema provides a standardized format for transforming unstructured language model outputs into symbolic representations that can be directly encoded within ACT-R.

Grounded in Frame Semantics, this approach assumes that meaning is best understood through structured relationships between entities within a conceptual frame. In this context, our schema functions as a minimal frame, capturing associations (e.g., group-attribute relationships) that reflect underlying sociocultural knowledge (Fillmore et al., 2006).

To ensure consistency and reduce noise, generation is constrained by setting a seed and controlled decoding parameters (i.e., a maximum generation length of 500 tokens, deterministic decoding, and *temperature* = 0.3) and enforcing a strict post-processing to retain only valid inputs. The generated sentences follow a similar structure to ConceptNet's “SurfaceText,” embedding the source entity, relation, and target entity in natural language form. For example, given an input word “ethnicity”, the model produces relevant sentences pertaining to the provided relations and generates statements such as “[Related to] Ethnicity relates to culture”. These sentence-like outputs are directly compatible with the downstream HDM pipeline, which expects a textual corpus as input. The specific prompt template used for the sentence generation is provided in Appendix A. Across all input terms, this process yields a corpus of several hundred relational sentences, forming the basis for subsequent chunk construction and cognitive simulation (Table 3).

**Table 3:** LLM corpora level stats.

Word	Total # of sen	Total # of raw words	Total # of pp words	Avg words per sen	Avg pp words per sen
All	728	3713	2328	5.1	3.2
Black	610	3047	1893	5.0	3.2
White	536	2635	1625	4.9	3.0

Specifically, the total number of words for each corpus, the total number of words after preprocessing for each corpus, the average words per sentence (raw), the average words per sentence (preprocessed).

### 3.5 Chunked inputs as HDM input

For this approach, we converted the inputs to a format similar to ACT-R's DM chunks. These dynamically generated chunks served as inputs to the model, enabling direct integration with the architecture's memory and retrieval mechanisms. Because of HDM integration, each chunk is additionally encoded with a time vector to ensure suitable retrieval. For the sources, we again used ConceptNet and LLM. For the ConceptNet source, we extracted the triples (start, relation, end) from the ConceptNet API. Every word had multiple triples based on different relations associated with the word. These triplets were added as chunks within HDM.

**Figure 5:**
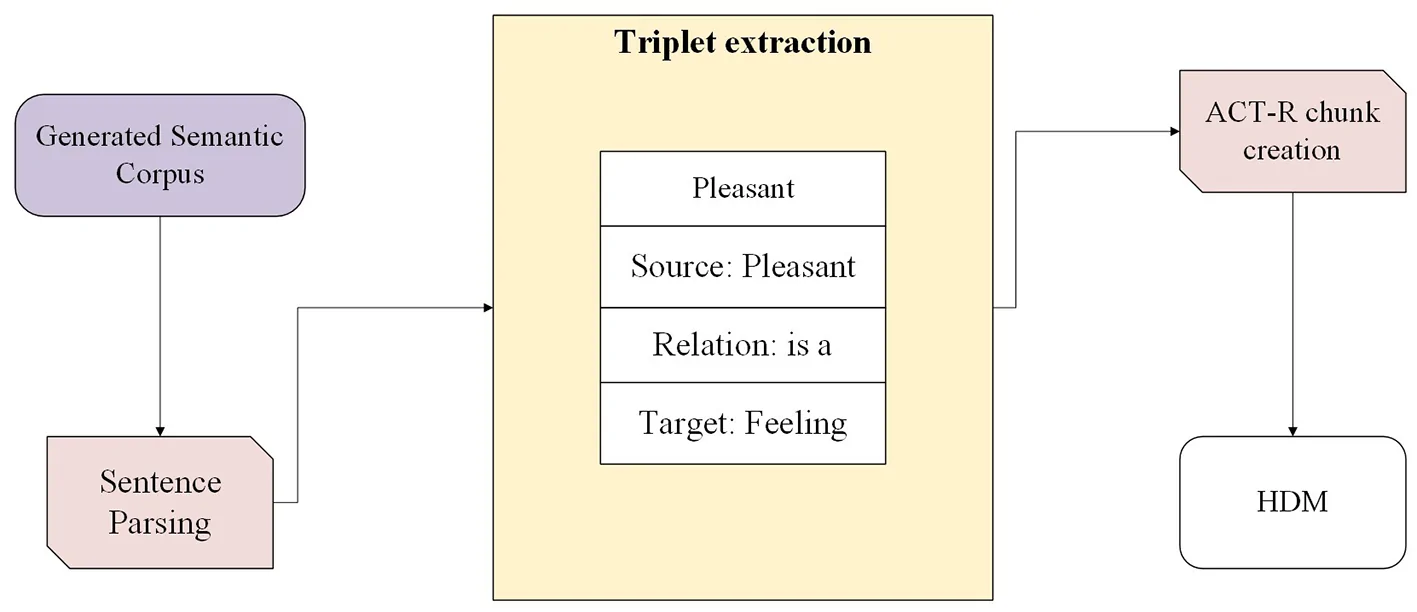
Conversion of LLM-generated corpus into ACT-R chunks through relation extraction and chunk argument construction, followed by encoding in HDM.

For the LLM-generated corpus, we parsed each sentence individually and extracted the triplets from the generated sentences based on the relation in [*relation*]*sentence* format. The generated relational triplets are then directly transformed into ACT-R-compatible declarative memory representations. Each triplet [*source, relation, target*] is mapped to a chunk consisting of corresponding slot-value pairs, consistent with ACT-R's representation of knowledge as structured symbolic units (Anderson et al., 2004) as outlined in Figure 5. This design choice is motivated by the need for compatibility between LLM outputs and cognitive architectures. While distributed representations such as word embeddings capture semantic similarity, they lack the explicit relational structure required for symbolic reasoning and cognitive simulation. By contrast, relational triplets align naturally with ACT-R's chunk schema, enabling seamless integration into declarative memory and subsequent evaluation through retrieval dynamics. This conversion also supports the analysis of association strength and bias, as relationships remain explicitly represented rather than implicitly encoded in vector space.

### 3.6 Human data

We collected data from human participants (N = 104) in an in-person user study conducted for a different research task. Following completion of the primary experimental task, participants completed the IAT, and these responses constitute the human dataset used for our analysis. The IAT was conducted using PsychoPy and was designed by Peirce et al. (2019). The block order and trials per block are mentioned in detail in subsection 3.2.

### 3.7 Design

We use the above-mentioned architecture pipeline (shown in Figure 2) to do a 2 x 2 x 3 comparison of various configurations. We compare 2 different methods of adding knowledge (Chunk based input, text corpus input), 2 different sources (LLM, ConceptNet), 3 different compositions of text corpus. We collect several metrics to analyze each configuration's performance on the IAT task. The key configurations are listed below:

1. Baseline: the *Baseline* configuration ran the IAT model without any input corpus, with the HDM integrated ACT-R, and this served as our baseline for further comparisons.
2. With text corpus: for both sources, LLM and ConceptNet, we used text generated from different seed sets of inputs.
2a. With All text corpus: for *Corpus All* configuration, we added a corpus of text generated from both of the knowledge sources with key index terms corresponding to words exposed to a user in the IAT task.
2b. With white text corpus: for *Corpus White* configuration, we added a corpus of text generated from both of the knowledge sources with key index terms related to White Race from the above seed terms list.
2c. With Black text corpus: for *Corpus Black* configuration, we added a corpus of text generated from both of the knowledge sources with key index terms related to Black Race from the above seed terms list.
3. Chunked inputs: for both sources, LLM and ConceptNet, we used chunked text as inputs to the model.
3a. With all chunks: for *Chunk All* configuration, we added chunked text generated from both of the knowledge sources with key index terms similar to text corpora inputs.
3b. With white chunks: for *Chunk White* configuration, we added chunked text generated from both of the knowledge sources with key index terms containing only White Race terms similar to text corpora inputs.
3c. With black chunks: for *Chunk Black* configuration, we added chunked text generated from both of the knowledge sources with key index terms containing only Black Race terms similar to text corpora inputs.

## 4 Results

Each configuration listed above was run 100 times, and we collected the metrics such as *d* − *score*, score for each stimulus, incorrect key presses, retrieval failures, noise in retrievals. We calculated *adjusted d* − *score* from *d* − *score* and incorrect keys pressed, and report them in the Table 4. Specifically, the Table 4 shows the mean and Standard error for 100 runs of all the configurations for *d* − *score*, *adjusted d* − *score*, accuracy, and test accuracy. We additionally included the results for the same IAT model (with the same parameters) without HDM and report them in Table 4 and in subsequent analyses as *DM*, as well as results from Human participants (*N* = 104) as *Human*.

**Table 4:** *d*-score, adjusted *d*-score, Total accuracy, and Test accuracy for all configurations.

Method	*d* − *score*	*adj d* − *score*	*Total* *Acc*	*Test* *Acc*
Human	−0.028 (0.068)	0.007 (0.07)	97.7 (1.05)	97.9 (1.04)
DM	0.002 (0.008)	0.002 (0.008)	91.3 (0.039)	99.8 (0.07)
Baseline	−0.010 (0.021)	−0.021 (0.026)	63.3 (1.22)	63.3 (1.22)
Text corpus				
*All* (CN)	0.006 (0.017)	0.016 (0.021)	81.9 (0.88)	81.8 (0.88)
*All* (LLM)	0.006 (0.017)	−0.015 (0.021)	79.7 (1.07)	79.5 (1.05)
*Black* (CN)	−0.000 (0.018)	0.015 (0.021)	80.7 (0.99)	80.5 (0.98)
*Black* (LLM)	0.019 (0.016)	0.006 (0.02)	78.9 (1.1)	78.9 (1.08)
*White* (CN)	0.016 (0.014)	0.06 (0.02)	80.2 (0.96)	80.2 (0.96)
*White* (LLM)	0.002 (0.02)	-0.009 (0.022)	80.0 (0.91)	79.9 (0.93)
Chunk				
*All* (CN)	−0.019 (0.051)	−0.04 (0.05)	33.2 (1.27)	32.5 (1.23)
*All* (LLM)	−0.013 (0.047)	−0.046 (0.049)	35.5 (1.31)	35.2 (1.29)
*Black* (CN)	−0.04 (0.06)	−0.066 (0.062)	31.8 (1.25)	31.8 (1.22)
*Black* (LLM)	−0.084 (0.054)	−0.056 (0.053)	34.4 (1.54)	34.0 (1.53)
*White* (CN)	−0.072 (0.057)	−0.034 (0.058)	32.9 (1.27)	32.7 (1.25)
*White* (LLM)	−0.003 (0.054)	−0.023 (0.056)	32.6 (1.23)	32.5 (1.22)

The first few rows indicate results from Human participants—Human, IAT model without HDM—DM, IAT model with HDM (without corpus)—baseline. Later block has text corpus for three different input word sets to HDM model, followed by chunked inputs to HDM model.

To calculate the *d* − *score*, we removed response times less than 300 ms, calculated the difference in mean response times between incongruent and congruent blocks, and divided it by the pooled standard deviation of correct-response latencies. This metric does not penalize for the wrong choices; so we use another metric that penalizes the model for incorrect responses (*adjusted d* − *score*). A penalty of 600 ms was added to the mean of that block when the incorrect response was chosen. The *Total Accuracy* was calculated as the percentage of correct retrievals for the total 70 stimuli, while the *Test Accuracy* was calculated as the percentage of correct retrievals for the total 40 test stimuli in the third and fifth blocks.

**Figure 6:**
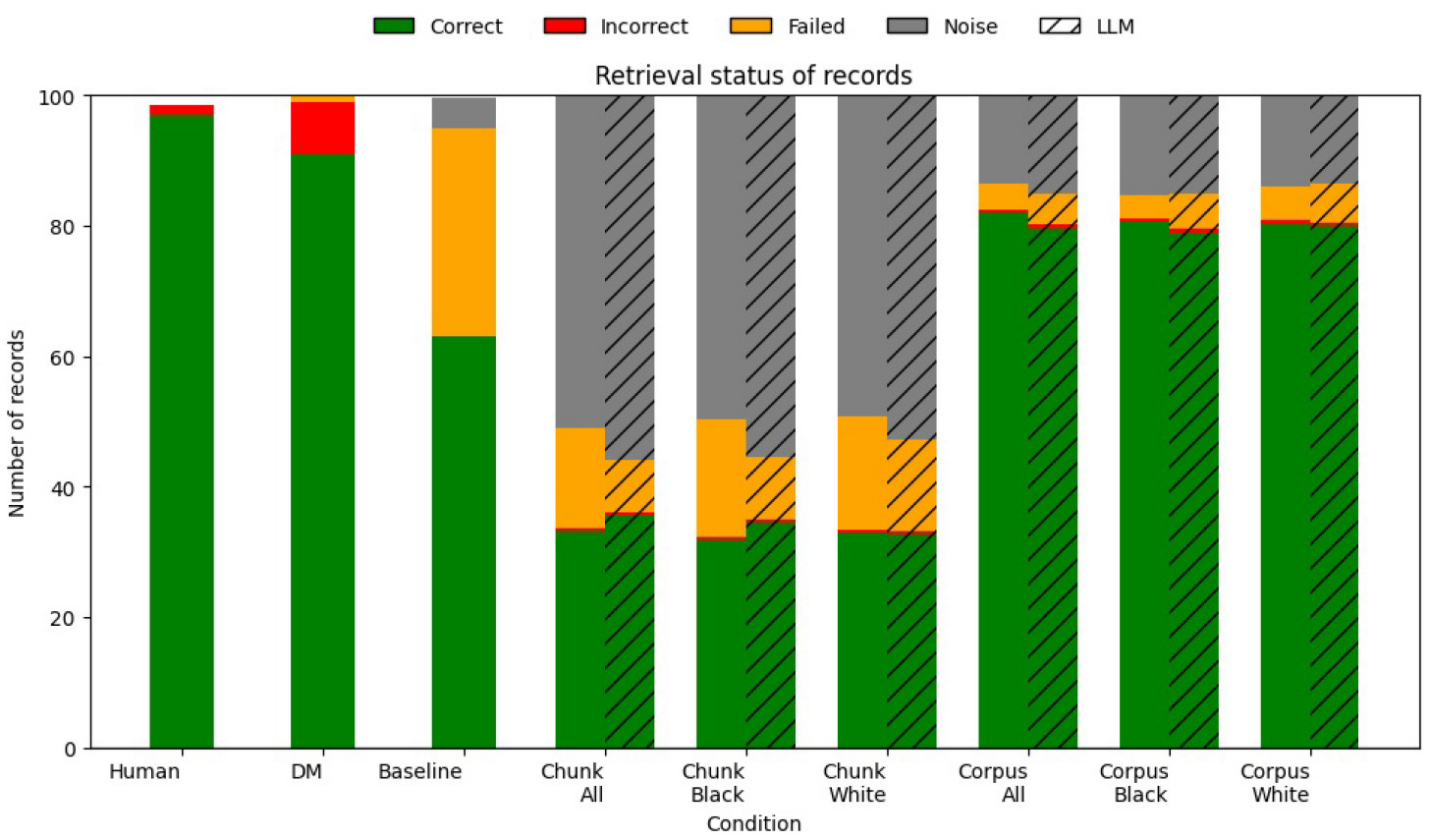
Comparing all chunk retrieval status for pure DM based data, human data, and all experiment configurations, with green representing a correct key pressed for successful retrieval, red representing an incorrect key pressed for successful retrieval, yellow representing chunk retrieval failure, and gray representing noise in retrieved chunk. The left bar represents data from ConceptNet, and the right (hatched) from LLM for the last 6 stacked bars.

Figure 6 compares how DM chunk retrieval might have affected accuracy. This indicates that the lower accuracy in *Baseline* is due to failed DM chunk retrieval, while all the chunked-corpora retrievals had higher noise. Among the chunked retrievals, failed chunk retrievals were higher in ConceptNet, while LLM-generated chunks had more noise in retrievals. The percentage of correct records retrieved was higher across all text corpus methods, with successful retrievals closer to pure *DM* based correct retrieval.

**Figure 7:**
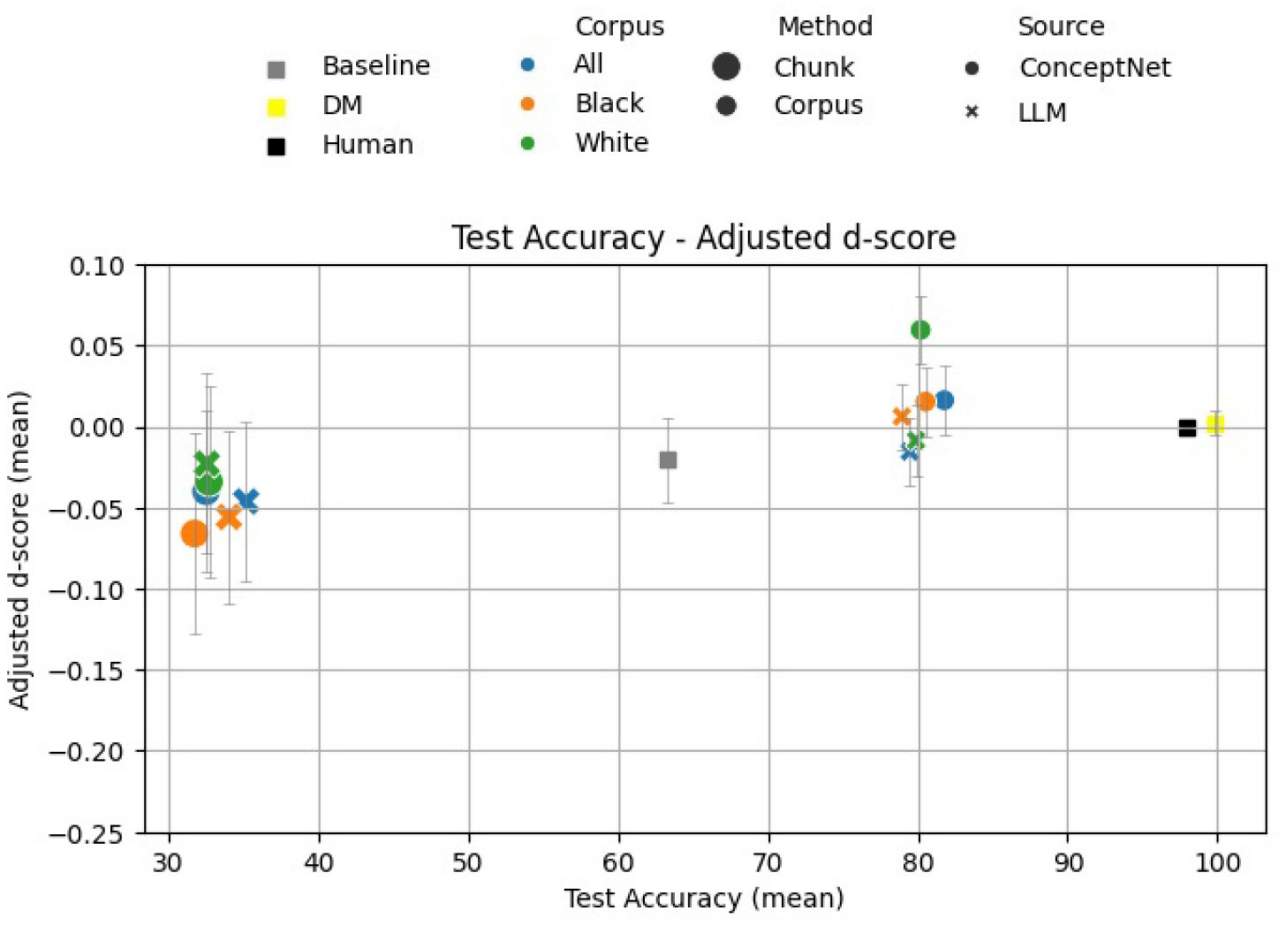
The adjusted d - score is plotted against test accuracy for all the configurations with sem bars. The left side cluster is dominated by chunked inputs, and the right cluster has all text input related configurations.

The Figure 7 plots the *adjusted d* − *score* mean along with the *Test Accuracy*. Based on this, we can clearly see that the accuracy of chunk-based methods is in the range (28,33) for both sources, while the accuracy of text corpus-based methods is between (79, 82) for both sources. Furthermore, ConceptNet showed a higher *adjusted d* − *scores* for the *White* corpus in both chunked and text corpus methods and the lowest *adjusted d* − *scores* for the *Black* corpus in both methods. While the LLM generated text corpus had a slightly higher *adjusted d* − *score* for *Black* and the lowest for the *All*, but the opposite was observed in chunked inputs. Further, *adjusted d* − *score* values were below 0 for chunked inputs and above 0 for text inputs, mostly. The Baseline *adjusted d* − *score* value, on the other hand, is slightly below 0 (−0.021) and has an accuracy of 63.3, which is between the chunked and text corpus methods. While both *Human* and pure *DM* data showed an adjusted d-score closer to 0, they had accuracies closer to 100.

Due to poor accuracy and higher error bars for chunked input, we performed a two-way ANOVA for knowledge sources within the text corpus and different corpus inputs, ignoring the chunked input data. This ANOVA revealed a statistically significant main effect of Source ($F=4.47,\;p<.05,\;\eta_{p}^{2}=0.007$), indicating differences in performance across sources. The main effect of corpus was not statistically significant ($F=0.72,\;p=.48,\;\eta_{p}^{2}=0.002$) nor was the Source x Corpus interaction, ($F=0.99,\;p=.369,\;\eta_{p}^{2}=0.003$).

## 5 Discussion

The HDM model begins with the random initialization of vectors, so words do not start with encoded biases. It then constructs memory vectors based on word associations in the corpus, allowing meaning to emerge from patterns of co-occurrence. This makes HDM useful for a race-based IAT because it can show whether race-related terms become more closely associated with positive or negative attributes based on the corpus itself. A positive *d* − *score* indicates an inclination toward a congruent test condition, which in our case is the association of White Race with pleasant terms, and a negative *d* − *score* indicates an inclination toward an incongruent test condition, which in our case is the association of Black Race with pleasant terms.

The chunked inputs for both sources (LLM and ConceptNet) reflected the expected *d* − scores, where the d-score for *White* is higher than *All*, which in turn is higher than *Black*. But overall, this resulted in lower accuracy than the corpora text method. Figure 6 shows that this is due to noise and not retrieval failure. The constructed HDM chunks include role-based slots like “Source,” “Target,” and “Relation,” which are common to all chunks. While this makes chunk construction easier, these chunks may not be compatible with our production rules, which map the expected chunk with the valence slot in our model. While this property emerges easily in text-based corpora due to increased co-occurrence, that may not be the case for role-based slots, which could introduce more noise and lead to erroneous chunk retrievals. To circumvent this, a better approach to chunked data input would be to use dynamically generated slots based on sentence structure rather than predefined role-based slots. However, such an approach could introduce a minor slowdown in chunk addition to HDM because the time vector is initialized for every new slot name within HDM for the chunks (Ray and Dancy, 2025), and requires a better algorithm to construct those slots, adding further steps to corpora generation. But this could improve the model's accuracy and could be considered in the future.

While, the relative ordering of LLM as source for *adjusted d* − *score* was *Black* > *White* > *All*, the ordering for ConceptNet was *White* > *All* >= *Black*. For ConceptNet, while the order of *d* − *scores* matched, the values were all positive in the corpus condition, indicating a predisposition to our congruent condition. Careful examination of the corpus generated by ConceptNet also shows a lack of statements for Black person-related terms and the “surfaceText” representing “anti-black” statements, which confirms the potential issues with the representation of Anti-blackness in ConceptNet, as noted by Dancy and Saucier, 2022. Moreover, the corpus generated by ConceptNet is deterministic, ensuring the same corpus is produced across all three conditions and accurately removing irrelevant sentences from *All* to generate *Black* and *White* sets.

For LLM, however, the adjusted d-scores reflected the opposite of the expectation. This points to a neutral-to-positive representation of Black person-related sentences, thereby suppressing harmful and stereotypical associations, as also reported by Ouyang et al., 2022. Another reason could be the presence of sentences pointing toward “racism” in the generated corpus, such as “White man can be racist”, “White man can be ignorant”. While problematic responses like “Black man is used for labor”, “Black man is used for entertainment” also exist in the same corpora, the number of positive sentences outnumber the problematic sentences substantially, potentially overcoming negative associations with stereotypes and causing higher d-scores for the “Black” corpus. This further suggests that both qualitative and quantitative properties of the corpus contribute directly to *d* − *scores*.

The parameters we chose for the Llama LLM resulted in less stochastic output. Though this made results more consistent, this lack of stochasticity may be less reflective of actual LLM use, reflecting a limitations of sorts (though the lack of transparency in many LLMs in use makes it difficult to understand how much this actually limited interpretation). Regardless of the stochasticity of the model output, using an open model does give an opportunity to better understand the probabilities of particular outputs given specific input. This opportunity for deeper understanding is important as it can be argued that token or sentence-level probabilities provide a clearer picture of model representation than simple prompt-output analysis (Hu and Levy, 2023). Given that we are developing architectural connections within a (more tractable) system, the use of an open model potentially allows for interesting analysis of the impact of changes in these probabilities of output, relations to latent structures in memory, and their downstream impacts on cognitive model behavior. Thus, combining the additional dimension of evaluation with a computational cognitive model trace gives an opportunity to detailed understanding of how the representations of concepts in LLMs may interact with cognitive processes to impact human behavior—this paves a pathway for a potentially powerful human-centric evaluation method, something previously suggested as a possible use of generative AI systems and cognitive architectures by Dancy and Workman (2023).

While the pipeline for loading the corpus and running the IAT task with HDM was the same and took approximately 45 s, corpus generation varied widely. Both sources required more RAM and GPU access to accelerate inference time or to build ConceptNet. For ConceptNet, the most RAM-intensive and time consuming task is downloading data and building it locally. However, once this setup is complete and the data is stored in a local database (such as PostgreSQL). To extract the corpus, the database server is initialized and queried via an API, which takes only a few minutes. For Llama, the setup involves downloading the pre-trained model weights, which can be several gigabytes in size. Loading the model requires significant VRAM, but once it is initialized, corpora can be generated directly through inference. This makes the process simpler in terms of the pipeline but more dependent on compute resources, and it can be challenging to run on edge devices unless the model is optimized through processes like quantization.

### 5.1 Limitations

A potential limitation with the IAT task is that the congruent condition is always presented before the incongruent condition. While an incongruent training block is included before testing to mitigate the remapping effects, we cannot entirely exclude the possibility that residual interference contributes to the measured latency differences. Therefore, switching the order of congruent and incongruent conditions in some of the experiments should be considered in future work.

Another limitation is the lack of detailed analysis of the generated corpora. Although additional analysis of the generated corpus could complement the IAT results, we believe that conventional corpus-level metrics, such as sentiment analysis or toxicity analysis, are insufficient to characterize the race-based associative structures relevant to this task. Developing or identifying evaluation methods that more faithfully capture these associations remains an important direction for our future work. Apart from these, some other future directions are mentioned in the next sub-section.

### 5.2 Future work

Building on the current results, in this section, we suggest possible future directions. We first examine incremental enhancements at each component level in our pipeline, discuss various alternatives, and then conclude by summarizing our contributions.

#### 5.2.1 Model size & quantization

Given the qualitative and quantitative impacts of corpus on *d* − *scores*, the first natural future direction would be to increase the number of sentences generated for each word and analyze the impacts. While this makes it harder to compare with ConceptNet, it could potentially reveal more patterns in the data and can be used to evaluate text generated from LLMs to assess the impact of problematic stereotypical associations.

Another future aim is to systematically investigate how different LLM characteristics influence the resulting *d* − *scores* and associated sociocultural patterns. In particular, we plan to evaluate models across varying scales to examine whether model size affects the strength or consistency of extracted associations. Additionally, we will compare different model families, such as different versions of Llama and Map-NEO (Zhang et al., 2024), to assess how architectural differences and training data composition impact bias expression. We also intend to explore the effects of model compression techniques, including quantization (Lin et al., 2024), to determine whether reduced precision alters the stability or interpretability of generated relations. By analyzing these factors, we seek to better understand how model design choices influence the extraction and representation of sociocultural knowledge within ACT-R.

#### 5.2.2 Knowledge graph extensions

Given the lighter coupling among individual components within the pipeline, it is easy to test different sources. Therefore, another future direction could be exploring different knowledge graphs such as $\text{ATOMIC}\begin{array}{c} 20\\ 20 \end{array}$ (Hwang et al., 2021), which provides rich inferential knowledge tuples about entities and events, TransOMCS (Zhang et al., 2020) that extended ConceptNet structure to automatic syntactic parsing of sentences from various web sources like Wikipedia, Yelp, and Reddit. Beyond single-hop triplets, another approach is to explore multi-hop data curation strategies that capture more complex relational reasoning by accounting for edge weights and semantically connected subsets of relations. Another direction involves a complex composition of single-hop nodes from the source node and the combination of multiple relations into coherent sentences, reinforcing the task-specific slot names and thereby increasing their activations. These potential future approaches will be incorporated into corpora generation, contributing to more robust corpora, strengthening the knowledge representation, and potentially resulting in higher accuracy for chunk-based inputs.

Another knowledge graph-related but slightly different approach could be using embeddings directly from knowledge graphs, for example, from ConceptNet Numberbatch (Speer et al., 2017a), to substitute the HDM initialization layer by providing dense, semantically grounded vectors that could eliminate the need for random orthogonal representations. Relational embedding models like HoLE (Nickel et al., 2016) could replace the HDM's vector space with a learned, relatively low-dimensional compositional embedding space in which entity interactions are inherently encoded via circular correlation. More broadly, adopting HoLE or related models shifts the system toward a fully relational, data-driven approach, improving on representational efficiency, but potentially sacrificing the cognitive plausibility.

#### 5.2.3 More complex cognitive models and tasks

Though the IAT is a useful task to initially explore for knowledge-level extensions that permit representations of sociocultural structures, given its direct connections and implications for social representations and the many human experiments carried out using the task, it remains a relatively simple task. This limits the potential impact of using a unified theory of cognition and cognitive architecture to implement the specific model. In the future, we will develop models of more complex tasks that will give us an opportunity to explore deeper connections between knowledge-level representations of social structures and cognitive processes.

In the domain of decision making, instance-based learning theory (IBLT) in dynamic decision making (Gonzalez et al., 2003) gives an opportunity to develop more interesting, complex models of decision making during various dynamic tasks. Additionally, HDM has been explored as a possible alternative memory system for IBLT in DDM (Kelly et al., 2020), albeit without a more generalized knowledge base such as the ones we test here. Thus, there is an opportunity expand this work to explore the impacts of sociocultural structures (in memory) on dynamic decision making, something especially important given the increasing use of opaque LLM-based systems and related AI systems in high impact environments (e.g., Domfeh and Dancy, 2025).

### 5.3 Conclusion

We presented a knowledge graph-based semantic vector system and an LLM-based system for adding more general knowledge bases to a computational cognitive architecture that uses scalable vector-based representations of symbolic concepts via Holographic Declarative Memory (HDM). HDM subsequently constructs memory vectors based on associative relationships between words in the corpus. In comparing the two systems, we found that while there were shown differences in the *adjusted d* − *score*, the only statistically significant difference was related to the source of knowledge, and not in the interaction between source and corpus. The emergence of these representations despite this random initialization would suggest, as expected, that it stems from statistical regularities in the knowledge environment (corpus, here) rather than from any built-in representational bias in initial HDM structures.

As we discussed in the previous section, the differences in adjusted *d* − score may be due to the propensity for newer LLMs to correct for particularly explicit forms of problematic racial associations, potentially impacting the downstream d-scores observed. The size of the different systems is also a notable difference between them. Given the environmental impacts of LLMs (e.g., Bender et al., 2021) and the classed, racialized impacts of environmental change (Verges, 2017), the development and use of these systems can be thought of as mediated by sociocultural structures (e.g., Workman and Dancy, 2026). Thus, there are several dimensions of consideration in thinking through both the theoretical and practical/material implications of employing either of these systems.

Ultimately, both systems present interesting opportunities for knowledge-level representation of sociocultural structures in computational cognitive models through cognitive architectures. Conversely, both systems also represent an opportunity to probe and evaluate those knowledge systems (i.e., ConceptNet and Llama 3.2 in this case) using more cognitively relevant and plausible methods that are more generalizable to actual human behavior. Though we present a relatively simple model here in the IAT, the results point to a unique and powerful opportunity to develop a deeper understanding of what the ubiquity of these (and related) systems, as potential knowledge sources through transactional memory, means for the propagation and bootstrapping of existing sociocultural knowledge and structures (Dancy and Saucier, 2022)—especially those that structures that uphold problematic, racialized representations of the human.

## Data Availability

The datasets presented in this study can be found in online repositories. The names of the repository/repositories and accession number(s) can be found below: THiCC Lab git repository–https://github.com/THiCC-Lab/paper-repos-pub.

## Appendix A: Prompt template used for LLM-based corpora generation

  SYSTEM PROMPT =
  You are a high-precision commonsense knowledge extraction system.
  Instructions:
  - Generate exactly 30 short factual statements.
  - Each statement must be less than 8 words.
  - Each statement must express one subject-relation-object fact.
  - Use only the listed relations.
  - Prefer obvious, stable commonsense facts.
  - Do not guess, if you are not confident for a relation report "don't Know".
  - Do not generate opinions, metaphors, stereotypes, or vague claims.
  - Do not repeat the same fact with different wording.
  - Output one line per fact.
  - Format each line as: [Relation] Sentence.
  
  USER PROMPT =
  Given a noun subject, generate short factual commonsense corpus statements using only the
   relations listed below.
  Subject: {text}
  Allowed relations: [ "Is A", "Related To", "Similar To", "Capable Of", "Part Of", "Used For",
   "Causes",
                       "Defined As", "Derived From", "Form Of", "Instance Of" ]
                  for each noun to generate factually accurate total 30 short, diverse and unique
                   sentences.
  Few-shot examples:
  Q: dog
  A:
  - [Is A] Dogs are animals.
  - [Instance Of] Dogs are mammals.
  - [Has A] Dogs have tails.
  - [Capable Of] Dogs can bark.
  - [Used For] Dogs can guard homes.
  - [At Location] Dogs live in homes.
  - [Causes] Don't know.
  Q: knife
  A:
  - [Used For] Knives cut food.
  - [Has A] Knives have blades.
  - [Has Property] Knives can be sharp.
  - [Related to] Don't know.
  - [Is A] Knives are tools.
  - [At Location] Knives are in kitchens.
  Q: cloud
  A:
  - [Causes] Clouds can cause shade.
  - [Related To] Clouds relate to weather.
  - [Has Property] Clouds can be gray.
  - [At Location] Clouds are in the sky.
  - [Part Of] Clouds are part of weather.
  Now generate accurate commonsense statements.
  Q: {text}
  A:
